# The dynamic pattern of end-tidal carbon dioxide during cardiopulmonary resuscitation: difference between asphyxial cardiac arrest and ventricular fibrillation/pulseless ventricular tachycardia cardiac arrest

**DOI:** 10.1186/cc9417

**Published:** 2011-01-11

**Authors:** Katja Lah, Miljenko Križmarić, Štefek Grmec

**Affiliations:** 1 Center for Emergency Medicine Maribor, Cesta proletarskih brigad 21, 2000 Maribor, Slovenia; 2 Department of Emergency Medicine, Faculty of Medicine University of Maribor, Slomškov trg 15, 2000 Maribor, Slovenia; 3 Faculty for Health Sciences University of Maribor, Žitna ulica 15, 2000 Maribor, Slovenia; 4 Department of Family Medicine, Poljanski nasip 58, Faculty of Medicine University of Ljubljana, 1000 Ljubljana, Slovenia

## Abstract

**Introduction:**

Partial pressure of end-tidal carbon dioxide (PetCO2) during cardiopulmonary resuscitation (CPR) correlates with cardiac output and consequently has a prognostic value in CPR. In our previous study we confirmed that initial PetCO2 value was significantly higher in asphyxial arrest than in ventricular fibrillation/pulseless ventricular tachycardia (VF/VT) cardiac arrest. In this study we sought to evaluate the pattern of PetCO2 changes in cardiac arrest caused by VF/VT and asphyxial cardiac arrest in patients who were resuscitated according to new 2005 guidelines.

**Methods:**

The study included two cohorts of patients: cardiac arrest due to asphyxia with initial rhythm asystole or pulseless electrical activity (PEA), and cardiac arrest due to arrhythmia with initial rhythm VF or pulseless VT. PetCO2 was measured for both groups immediately after intubation and repeatedly every minute, both for patients with or without return of spontaneous circulation (ROSC). We compared the dynamic pattern of PetCO2 between groups.

**Results:**

Between June 2006 and June 2009 resuscitation was attempted in 325 patients and in this study we included 51 patients with asphyxial cardiac arrest and 63 patients with VF/VT cardiac arrest. The initial values of PetCO2 were significantly higher in the group with asphyxial cardiac arrest (6.74 ± 4.22 kilopascals (kPa) versus 4.51 ± 2.47 kPa; *P *= 0.004). In the group with asphyxial cardiac arrest, the initial values of PetCO2 did not show a significant difference when we compared patients with and without ROSC (6.96 ± 3.63 kPa versus 5.77 ± 4.64 kPa; *P *= 0.313). We confirmed significantly higher initial PetCO2 values for those with ROSC in the group with primary cardiac arrest (4.62 ± 2.46 kPa versus 3.29 ± 1.76 kPa; *P *= 0.041). A significant difference in PetCO2 values for those with and without ROSC was achieved after five minutes of CPR in both groups. In all patients with ROSC the initial PetCO2 was again higher than 1.33 kPa.

**Conclusions:**

The dynamic pattern of PetCO2 values during out-of-hospital CPR showed higher values of PetCO2 in the first two minutes of CPR in asphyxia, and a prognostic value of initial PetCO2 only in primary VF/VT cardiac arrest. A prognostic value of PetCO2 for ROSC was achieved after the fifth minute of CPR in both groups and remained present until final values. This difference seems to be a useful criterion in pre-hospital diagnostic procedures and attendance of cardiac arrest.

## Introduction

Capnometry and capnography have gained a crucial role in monitoring critically ill patients in the pre-hospital setting [1-5]. They can be used as a detector for correct endotracheal tube placement, to monitor the adequacy of ventilation, ensure a proper nasogastric tube placement, recognize changes in alveolar dead space, help describe a proper emptying pattern of alveoli, help estimate the deepness of sedation and relaxation in critically ill, help in diagnostics of severe pulmonary embolism, and can be used in cardiac arrest patients as a prognostic determinant of outcome and in monitoring the effectiveness of cardiopulmonary resuscitation (CPR) [6-11]. In our previous study [12], we found that initial values of partial pressure of end-tidal carbon dioxide (PetCO2) in asphyxial arrest were significantly higher than in ventricular fibrillation/pulseless ventricular tachycardia (VF/VT) arrest. In asphyxial arrest there was also no significant difference in initial values of PetCO2 in patients with and without return of spontaneous circulation (ROSC). In asphyxial arrest the initial values of PetCO2 cannot be used as a prognostic factor of outcome of CPR, as they can be in VF/VT arrest [13-16]. This difference, together with other criteria, can therefore be useful for differentiating between the causes of cardiac arrest in the pre-hospital setting [17]. In this study we sought to evaluate the pattern of PetCO2 changes in cardiac arrest caused by VF/VT and asphyxial cardiac arrest in patients who were resuscitated according to new 2005 guidelines [18-20].

## Materials and methods

This prospective observational study was conducted at the Center for Emergency Medicine, Maribor, Slovenia. To facilitate a true comparison, the design of this study was identical to our first one. Patients constitute two cohorts. The study was approved by the Ethical Board of the Ministry of Health, which granted waiver of informed consent (victims of cardiac arrest). Patients who regained consciousness or their relatives were informed after enrollment.

The first cohort included patients who suffered from cardiac arrest due to asphyxia. The causes of asphyxia were: asthma, severe acute respiratory failure, tumor of the airway, suicide by hanging, acute intoxication, pneumonia and a foreign body in the airway. The definitive cause of cardiac arrest was confirmed in the hospital by further diagnostic and/or pathology reports (post mortem). The initial rhythm seen on the monitor for all the patients in this group was either asystole or pulseless electrical activity. We excluded patients in severe hypothermia (core temperature <30°C) and patients with incomplete measurements of PetCO2 in the first 10 minutes of CPR.

The second group included patients who suffered from primary cardiac arrest (acute myocardial infarction or malignant arrhythmias). The definitive cause of cardiac arrest was confirmed in the hospital by further diagnostic and/or pathology reports (post mortem). The initial rhythm seen on the monitor for all the patients in this group was either VF or pulseless VT. We excluded patients in severe hypothermia (core temperature <30°C) and patients with incomplete measurements of PetCO2 in the first 10 minutes of CPR.

The inclusion/exclusion criteria for both groups are presented in Table 1.

**Table 1:** Inclusion/exclusion criteria for both groups

**Inclusion criteria**:	VF/VT group	Asphyxia group
Initial rhythm	VF/VT	Asystole or PEA
Age	>18 years	>18 years
Core temperature	>30°C	>30°C
Measurement of PetCO2 values	Every minute in the first 10 minutes after intubation	Every minute in the first 10 minutes after intubation
Aetiology	Confirmed acute myocardial infarction and/or primary VF/VT (electrocardiogram, enzymes, electrophysiological studies)	Confirmed asphyxial cause (acute asthma attack, severe acute respiratory failure, tumor of the airway, suicide by hanging, acute intoxication, aspiration, foreign body in the airway)
Exclusion criteria:		
CPR procedures	Successful defibrillation in the first cycle	VF or pulseless VT as the initial rhythm on the monitor
Aetiology	Acute myocardial infarction with asystole or PEA as the initial rhythm (autopsy or additional investigations in the hospital)	Acute myocardial infarction as a cause of arrest (autopsy or additional investigations in the hospital)

Resuscitation procedures were performed by an emergency medical team (emergency medical physician and two emergency medical technicians or registered nurses) in accordance with 2005 ERC Guidelines. For management of VF and pulseless VT, direct-current counter-shocks were delivered by means of standard techniques.

PetCO2 measurements were made by infrared sidestream capnometer (BCI Capnocheck Model 20600A1; BCI International, Waukesha, WI, USA). Measurements for both groups were made immediately after endotracheal intubation (first measurement) and then repeatedly every minute continuously. Endotracheal intubation was performed at the beginning of CPR. Ventilation was performed by mechanical ventilator (6 ml/kg, 10 breaths/minute; Medumat Standard Weinmann, Hamburg, Germany). The carbon dioxide (CO2) cuvette was located in a connector between the mechanical ventilator and the endotracheal tube; it was applied to the endotracheal tube before the intubation.

We obtained the initial (first measurement after intubation), average after one minute of CPR, and final (measurement at admission to the hospital or discontinued CPR) value of PetCO2 for both groups. We also decided to obtain values of PetCO2 after two, three, five and ten minutes of CPR. We performed the same procedures for the patients with and without ROSC.

ROSC is defined as a return of spontaneous circulation or as palpable peripheral arterial pulse and measurable systolic arterial pressure. In the present study ROSC represented hospitalized patients.

All the data were collected in Microsoft Excel tables. The paired Student *t*-test was used to compare initial and subsequent PetCO2 values for each subject. For other parameters, both groups were compared by Student *t-*test and χ^2 ^test. Continuous variables are described as the mean ± standard deviation. *P *< 0.05 was considered significant.

## Results

**Figure 1:**
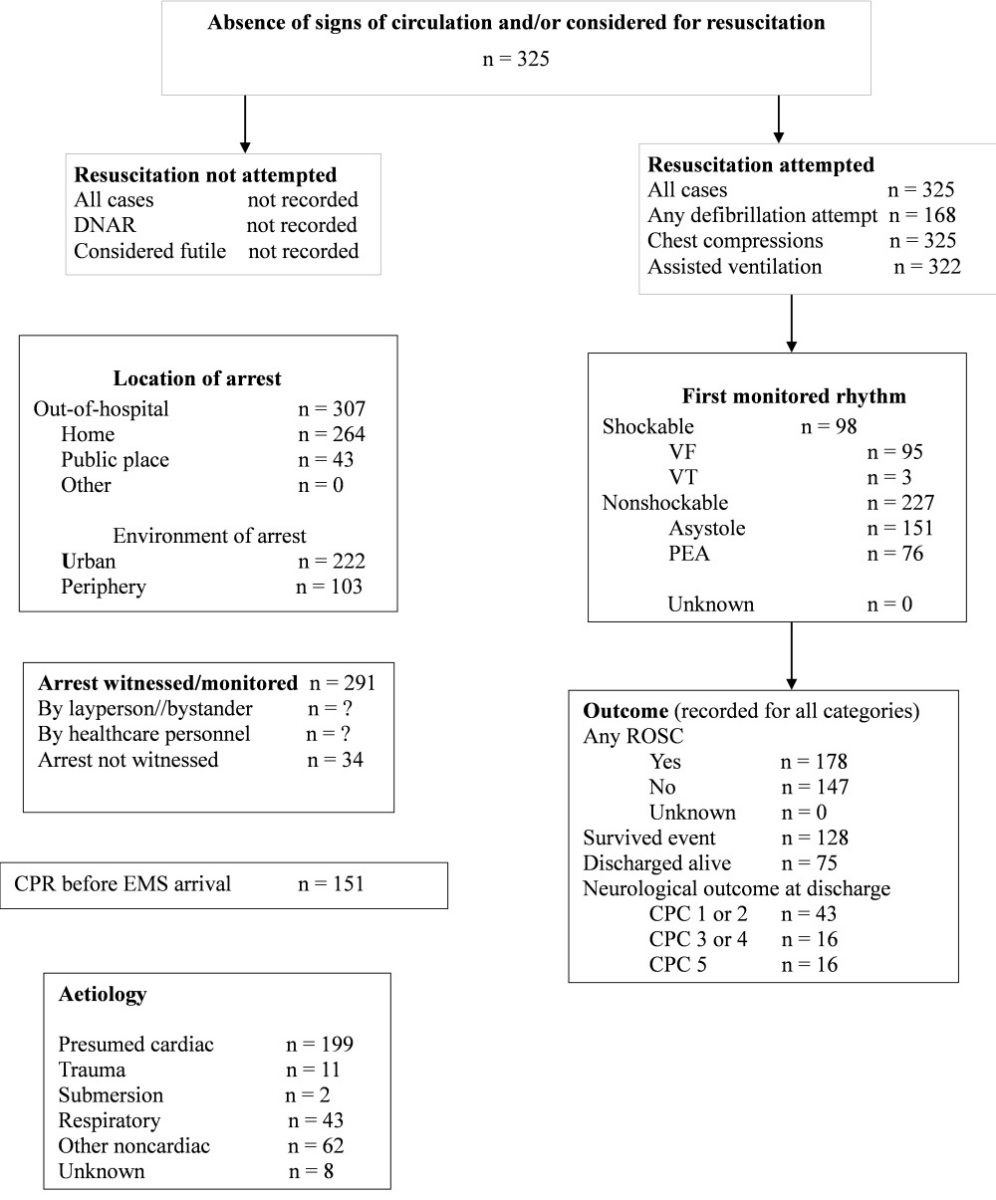
**All cardiac arrests placed in the Utstein template**. CPC, cerebral performance categories; DNAR, do not attempt resuscitation; EMS, emergency medical service; PEA, pulseless electrical activity; ROSC, return of spontaneous circulation; VF, ventricular fibrillation; VT, ventricular tachycardia.

Between June 2006 and June 2009 resuscitation was attempted in 325 patients (ROSC was 55%, admission to hospital 40% and discharge rate was 23%). The study environment, the pre-hospital environment and characteristics of cardiac arrest are presented in Figure 1 as an Utstein style report. Of those who received CPR, 211 were excluded; 8 patients had cardiac arrest of unknown aetiology, 11 patients had cardiac arrest precipitated by trauma and 192 failed inclusion criteria or met exclusion criteria, hence, leaving 51 patients with asphyxial cardiac arrest and 63 patients with primary cardiac arrest. Demographic and clinical characteristics for both groups are presented in Table 2.

**Table 2:** Demographic and clinical characteristics for both groups of patients

	Primary cardiac arrest - VF/VT (*n* = 63)	Asphyxial cardiac arrest (*n* = 50)	*P-*value
Age (years)	62.6 + 11.6	59.45 + 19.04	0.497¹
Gender(Male/Female)	50/13	27/23	0.003²
Response time (minute)ª	7.12 + 4.5	6.64 + 4.47	0.858¹
Witnessed arrest (Yes/no)	60/3	43/7	0.050²
Resuscitation by medical team (min)	29.7 + 17.2	28.2 + 21.3	0.58¹
ROSC (yes/no)	45/18 = 71%	27/24 = 53%	0.175²
Discharged from ICU (yes/no)	40/23 = 63%	20/30 = 39.2%	0.011²
Discharged alive (yes/no)	25/38 = 39.6%	9/41 = 17.6%	0.009²
CPC 1 to 2 (yes/no)	17/46 = 26.9%	5/45 = 9.8%	0.04²
Average number ob PetCO2 observations	9 (between 3 in 19)	9 (between 2 in 22)	0.312²

CPC cerebral performance category; ICU, intensive care unit; PetCO2 partial pressure of end-tidal carbon dioxide; ROSC return of spontaneous circulation

ª Time elapsed between the 112 call and the arrival of emergency medical team to the patient.

¹ Student *t-*test.

² χ^2 ^test.

The causes of asphyxial cardiac arrest were acute asthma attack (15 cases), severe acute respiratory failure (15 cases), tumor of the airway (3 cases), suicide by hanging (3 cases), pneumonia (4 cases), acute intoxication (8 cases), and foreign body in the airway (3 cases).

**Figure 2:**
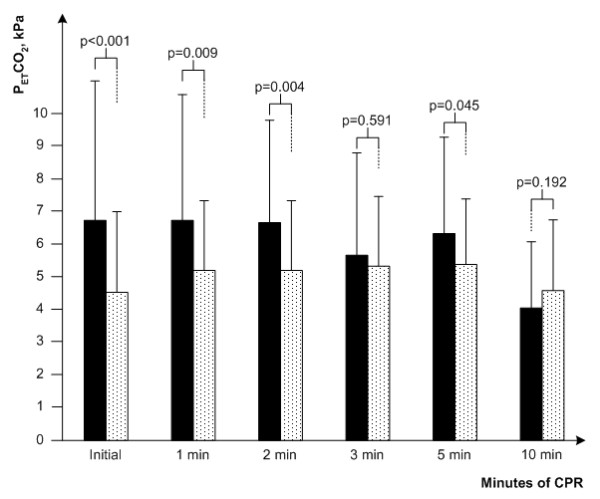
**End-tidal pCO2 during cardiopulmonary resuscitation in all patients included in study**. All patients: asphyxial cardiac arrest (black bar), primary cardiac arrest (dotted bar). CPR, cardiopulmonary resuscitation; PetCO2, partial pressure of end-tidal carbon dioxide.

The values of PetCO2 for all patients are presented in Figure 2. The initial values of PetCO2 were significantly higher in the group with asphyxial cardiac arrest (6.74 ± 4.22 kilopascals (kPa) versus 4.51 ± 2.47 kPa; *P *= 0.004). The values of PetCO2 remained significantly higher until the third minute of CPR, by then there was no remaining significant difference between the groups (5.63 ± 3.11 kPa versus 5.36 ± 2.17 kPa; *P *= 0.654). There is also no significant difference between the groups at the final values of PetCO2 (5.96 ± 2.18 kPa versus 5.12 ± 1.57 kPa; *P *= 0.105).

**Figure 3:**
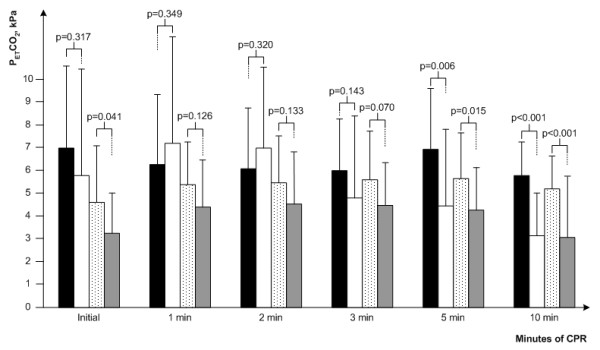
**End-tidal pCO2 during cardiopulmonary resuscitation regarding aetiology of cardiac arrest and outcome**. PetCO2 during cardiopulmonary resuscitation. Asphyxial with ROSC (black bar), asphyxial without ROSC (white bar), VF/VT with ROSC (dotted bar) and VF/VT without ROSC (gray bar). Data are presented as mean values with one standard deviation. *P*-values were calculated by unpaired *t*-test for each time period and show above bars. CPR, cardiopulmonary resuscitation; PetCO2, partial pressure of end-tidal carbon dioxide; ROSC, return of spontaneous circulation.

We also compared patients with and without ROSC within both groups. The values of PetCO2 for both groups according to ROSC are presented in Figure 3.

In the group with asphyxial cardiac arrest the initial values of PetCO2 did not show significant difference when we compared patients with and without ROSC (6.96 ± 3.63 kPa versus 5.77 ± 4.64 kPa; *P *= 0.313). We confirmed significantly higher initial PetCO2 values for those with ROSC in the group with primary cardiac arrest (4.62 ± 2.46 kPa versus 3.29 ± 1.76 kPa; *P *= 0.041). The significant difference in PetCO2 values for those with and without ROSC was achieved after the fifth minute of CPR in both groups (asphyxial arrest: 6.09 ± 2.63 kPa versus 4.47 ± 3.35 kPa; *P *= 0.006; primary arrest: 5.63 ± 2.01 kPa versus 4.26 ± 1.86; *P *= 0.015) and remained present until final values of PetCO2 (asphyxial arrest: 5.87 ± 2.14 kPa versus 0.55 ± 0.49 kPa; *P *< 0.001, primary arrest: 4.99 ± 1.59 kPa versus 0.96 ± 0.39 kPa; *P *< 0.001). In all patients with ROSC the initial PetCO2 was again higher than 1.33 kPa.

After one minute of CPR we observed no significant difference in those with and without ROSC in both groups (asphyxial arrest: 6.26 ± 3.03 kPa versus 7.31 ± 4.69 kPa; *P *= 0.345, primary arrest: 5.35 ± 2.18 kPa versus 4.42 ± 2.09 kPa; *P *= 0.134). After two minutes (asphyxial arrest: 6.07 ± 2.66 kPa versus 6.96 ± 3.54; *P *= 0.316, primary arrest: 5.48 ± 2.10 kPa versus 4.56 ± 2.31 kPa; *P *= 0.351) and three minutes (asphyxial arrest: 6.08 ± 2.29 kPa versus 4.82 ± 3.64 kPa; *P *= 0.143, primary arrest: 5.56 ± 2.14 kPa versus 4.49 ± 1.86 kPa; *P *= 0.070) of CPR there still was no significant difference among those with and without ROSC.

We also observed a significant improvement in intensive care unit (ICU) survival rates for both groups. When we compared the first and this study, a significant difference was achieved for patients who suffered from asphyxial cardiac arrest (7/37 (16%) versus 20/31 (39.2%); *P *= 0.02) and for those who suffered from VF/VT cardiac arrest (38/103 (27%) versus 40/23 (63.5%); *P *< 0.01).

## Discussion

In this study, which was conducted according to ERC 2005 Guidelines, we confirmed higher values of initial PetCO2 in asphyxial cardiac arrest than in primary cardiac arrest. The high initial values of PetCO2 in asphyxial cardiac arrest did not have a prognostic value for ROSC.

The 2005 ERC Guidelines differ from the 2000 ERC Guidelines mainly in a shift from primary rhythm-based management of cardiac arrest to a focus on neurological outcomes. The guidelines in the second study period are intensely focused on cardiac massage; the compressions:ventilation ratio is 30:2, the hands-off time is mitigated and if the access time is longer than three minutes, there are first two minutes of CPR before the first defibrillation. Only a single shock is administrated instead of a three-shock sequence [21-26].

Nevertheless, the general pattern of PetCO2 changes remains the same. In asphyxial cardiac arrest the initial values are high, and do not have prognostic value for ROSC, then decrease later in CPR and increase again in patients with ROSC [27,28]. In primary cardiac arrest the initial values are significantly higher in patients with ROSC. The difference from the first study [12] is shown in the first and the second minute of CPR. In this study the significant difference between the two groups remains until the third minute of CPR. This may be a result of a higher emphasis on cardiac massage, which causes more CO2 to be shifted from a peripheral compartment. Both studies were conducted in out-of -hospital environments, which meant longer access times and different first approaches. In the first study we started with rhythm recognition in order to defibrillate as soon as possible, whereas in this study we started with cardiac massage immediately after cardiac arrest was recognized. This probably leads to more intense shipment of CO2 from the peripheral compartment, which then causes values of PetCO2 to remain higher for a longer time. The pattern is restored after the third minute of CPR, when the values decrease and later increase again only in patients with ROSC. The significant difference in PetCO2 values (and restart of a prognostic value of PetCO2) for those with and without ROSC was achieved after five minutes of CPR in both groups and remained present until final values of PetCO2. In both studies the initial PetCO2 values for all patients with ROSC were higher than 1.33 kPa.

In the second study, where resuscitation was conducted according to the 2005 ERC Guidelines, we also observed a significant increase in ICU survival rates in both groups.

Assisted ventilation can be postponed in VF/VT cardiac arrest [29,30]. On the other hand, quick intervention with assisted ventilation in the field can be life saving in asphyxial cardiac arrest [31-33]; therefore, it is important to be able to recognize the cause of cardiac arrest.

### Limitations

This study has some limitations. First, our sample size is reasonable (rigorous inclusion and exclusion criteria), but a larger cohort may have afforded the opportunity for complete subgroup analysis. Second, PetCO2 is only an indirect measurement of cardiac out-put and a two-compartment model of CO2 [12]. In the next study we should include point-of-care bedside blood gas analysis and point-of-care ultrasound in the field. Third, better results in the second study are the results of the improvement of skills, methods of CPR (new guidelines) and bystander CPR.

## Conclusions

The dynamic pattern of PetCO2 values during out-of-hospital CPR shows higher values of PetCO2 in the first two minutes of CPR in asphyxial and prognostic value of initial PetCO2 only in primary VF/VT cardiac arrest. The prognostic value of PetCO2 for ROSC was achieved after the fifth minute of CPR in both groups and remained present until the final values.

The values of PetCO2 seem to be useful in differentiating causes of cardiac arrest in the pre-hospital setting.

## Key messages

• Initial values of PetCO2 are higher in asphyxial cardiac arrest than in primary cardiac arrest.

• Initial values of PetCO2 in asphyxial cardiac arrest do not have a prognostic value for resuscitation outcome.

• The prognostic value of PetCO2 for ROSC was achieved after the fifth minute of CPR in both groups and remained present until the final values.

• The values of PetCO2 seem to be useful in differentiating the causes of cardiac arrest in a pre-hospital setting.

## Abbreviations

ARDS: acute respiratory distress syndrome; CO2: carbon dioxide; CPC: cerebral performance categories; CPR: cardiopulmonary resuscitation; EMS: emergency medical service; ERC: European Resuscitation Council; ICU: intensive care unit; kPa: kilopascals; PEA: pulseless electrical activity; PetCO2: partial pressure of end-tidal carbon dioxide; ROSC: return of spontaneous circulation; VT/VF: ventricular fibrillation/pulseless ventricular tachycardia.

## Competing interests

The authors declare that they have no competing interests.

## Authors' contributions

LK was involved in the writing of the study protocol, collected the data, analysed and interpreted the data and wrote the draft of the manuscript. MK was involved in designing the study protocol and statistical analysis and interpreted the data. SG was involved in designing and writing the study protocol, analysed and interpreted the data and made comments on the draft of the manuscript.
